# Efforts by the Coalition to End Social Isolation and Loneliness

**DOI:** 10.1093/geroni/igab046.673

**Published:** 2021-12-17

**Authors:** Andrew MacPherson

**Affiliations:** Healthsperien, Washington, District of Columbia, United States

## Abstract

Established in the Fall 2018 and based in Washington, D.C., the Coalition to End Social Isolation & Loneliness brings together dozens of national organizations including consumer groups, community-based organizations, health plans, mental and behavioral health organizations, health care innovators, and many others to lead a multi-stakeholder dialogue to address the crisis of social isolation and loneliness in America. The Coalition focuses on three major areas to achieve this goal: Disseminating research findings, developing and advocating for federal and state legislative and regulatory policy interventions, and leading public awareness events in Washington, D.C. and across the nation. The COVID-19 pandemic has greatly accelerated efforts to engage Congress and the Executive Branch on a range of federal policy priorities, including leveraging and advancing social services and supports, supporting health care delivery to support those who are socially isolated and/or lonely, and advancing federally-funded research initiatives.

